# Assessment of the Angolan (CHERRT) Mobile Laboratory Curriculum for Disaster and Pandemic Response

**DOI:** 10.5811/westjem.2020.4.47385

**Published:** 2020-04-13

**Authors:** Michael D. Owens, Michael L. Lloyd, Tyler M. Brady, Robin Gross

**Affiliations:** * Naval Medical Center Portsmouth, Portsmouth, Virginia; † Integrated Research Facility, National Institute of Allergy and Infectious Diseases, National Institutes of Health, Fort Detrick, Frederick, Maryland

## Abstract

**Introduction:**

As of April 5, 2020, the World Health Organization reported over one million confirmed cases and more than 62,000 confirmed coronavirus (COVID-19) deaths affecting 204 countries/regions. The lack of COVID-19 testing capacity threatens the ability of both the United States (US) and low middle income countries (LMIC) to respond to this growing threat, The purpose of this study was to assess the effectiveness through participant self-assessment of a rapid response team (RRT) mobile laboratory curriculum

**Methods:**

We conducted a pre and post survey for the purpose of a process improvement assessment in Angola, involving 32 individuals. The survey was performed before and after a 14-day training workshop held in Luanda, Angola, in December 2019. A paired t-test was used to identify any significant change on six 7-point Likert scale questions with α< 0.05 (95% confidence interval).

**Results:**

All six of the questions – 1) “I feel confident managing a real laboratory sample test for Ebola or other highly contagious sample;” 2) “I feel safe working in the lab environment during a real scenario;” 3) “I feel as if I can appropriately manage a potentially highly contagious laboratory sample;” 4)“I feel that I can interpret a positive or negative sample during a suspected contagious outbreak;” 5) “I understand basic Biobubble/mobile laboratory concepts and procedures;” and 6) “I understand polymerase chain reaction (PCR) principles” – showed statistical significant change pre and post training. Additionally, the final two questions – “I can more effectively perform my role/position because of the training I received during this course;” and “This training was valuable” – received high scores on the Likert scale.

**Conclusion:**

This Angolan RRT mobile laboratory training curriculum provides the nation of Angola with the confidence to rapidly respond and test at the national level a highly infectious contagion in the region and perform on-scene diagnostics. This mobile RRT laboratory provides a mobile and rapid diagnostic resource when epidemic/pandemic resource allocation may need to be prioritized based on confirmed disease prevalence.

## INTRODUCTION

### Background

As of April 5, 2020, the World Health Organization (WHO) reported 1,133,758 confirmed cases and 62,784 confirmed coronavirus (COVID-19) deaths affecting 204 countries/regions.^1^ It is recognized that the lack of COVID-19 testing capacity threatens the ability of both the United States (US) and low middle income countries (LMIC) to respond to this growing threat.^2^ For comparison, the 2014 Ebola epidemic in Western Africa (Liberia, Sierra Leone, Guinea, and Nigeria) infected tens of thousands of individuals and claimed more than 11,000 lives, with a case fatality rate of approximately 60%.^3^,^4^

There is growing evidence that this current outbreak is more widespread than reported due to the lack of laboratory capacity and resources.^5^ This parallels the experiences identified in “After Action Reports” and lessons learned during the 2014 Western Africa Ebola epidemic. However, COVID-19 is now a pandemic affecting multiple LMICs and the US whose current laboratory capacity is limited. 2

A mobile laboratory (bioBUBBLE, Inc., Fort Collins, CO) using GeneXpert (Cepheid Inc, Sunnyvale, CA) technology to conduct reverse transcription polymerase chain reaction (RT-PCR) was deployed during the 2014 West Africa Ebola outbreak and again in the 2017 Democratic Republic of Congo (DRC) Ebola outbreak.^6^ This field-deployable diagnostic tool provided results in as little as 90 minutes. RT-PCR is a laboratory technique combining reverse transcription of ribonucleic acid to deoxyribonucleic acid (DNA) and the amplification of disease-specific DNA targets using the polymerase chain reaction (PCR). In acute respiratory infections, RT-PCR is used to detect viruses from respiratory secretions. The use of this technology to develop a simple, rapid, and robust detection capability with minimal training and lab experience or infrastructure has been demonstrated during previous international health emergencies such as severe acute respiratory syndrome (SARS).^5^

On March 21, 2020, it was reported that the US Federal Drug Administration approved the first rapid point-of-care COVID-19 test capable of delivering results in under an hour.^7^ This test kit involves taking a nasopharyngeal swab and can be done in an office, clinic, or a mobile lab in about 45 minutes. Administering the test does not require any specialty training other than what was provided within the curriculum, and the lab is capable of running 24 hours a day/seven days a week.

This current global outbreak presents challenges to local, regional, and national medical communities to mitigate the current pandemic. A global response involving logistical, epidemiological, public health, and medical interventions may slow and contain the further spread of this contagion. Employing a mobile lab with biocontainment capability and a rapid, automated diagnostic test in regions where on-site diagnostics may be of benefit allows for a focused response and resource distribution by rapidly identifying positive cases.

One of the mitigation and response strategies learned during the 2014 Ebola epidemic includes rapid response teams that are trained, prepared, and mobilized immediately when a suspect case is identified. A strategy of Rapid Isolation and Treatment of Ebola using the Liberia Ministry of Health and Social Welfare (MOHSW), supported by the US Centers for Disease Control and Prevention (CDC), WHO, and other agencies in Liberia began to respond systematically to suspected cases in remote areas in August 2014.^3^ This rapid response concept, when instituted in later outbreaks, was one of the factors that helped contain the Ebola virus disease (EVD) outbreak in the DRC in May 2017. Teams that are competently and confidently trained in RT-PCR, personal protective equipment (PPE) protocols, and mobile laboratory capabilities provide a valuable resource by confirming cases quickly and efficiently. Follow-up interventions are then deployed in a manner such that treatment, isolation/quarantine efforts, and other response and mitigation efforts are more effective and focused.

**Population Health Research Capsule**

What do we already know about this issue?

The African Ebola outbreaks and current COVID-19 pandemic affirm that rapid and accurate diagnostics influence response and outcomes.

What was the research question?

We conducted an assessment of a mobile lab curriculum for disaster and pandemic response based on current models.

What was the major finding of the study?

This curriculum provided the Angola Community Health Emergency Rapid Response Team with the confidence to respond to a disaster/pandemic at the national level.

How does this improve population health?

Rapid and accurate diagnostic confirmation of a Public Health Emergency of International Concern using a mobile lab improves response and mitigates further spread of a contagion.

The nation of Angola, due to its proximity to EVD-endemic areas and its own experiences with Marburg and 2017 yellow fever epidemics, created a Community Health Emergency Rapid Response Team (CHERRT) sponsored by the national military and Ministry of Health (MOH). These Angolan RRT members were trained for two weeks in December 2019 by the US Navy and experts from the National Institutes of Health (NIH). The first week of training included tabletop scenarios, individual break-out sessions, didactic lectures, hands-on training with equipment and PPE, and patient scenarios. The second week of training focused on RT-PCR, laboratory techniques, diagnostics, and use of the mobile lab and rapid diagnostic on-site testing. A pre- and post-training survey was completed by the participants, to include self-assessments of their perceived ability to perform RT-PCR diagnostics and work with the mobile lab.

## OBJECTIVES

The purpose of this study was to assess the effectiveness through participant self-assessment of a RRT mobile lab curriculum based on the WHO Ebola Virus Disease Consolidated Preparedness Checklist, Revision 1^8^ and the mobile lab training curriculum developed by the NIH. Using a pre-post confidential questionnaire with eight 7-point scale Likert questions and an open-ended comments section, we assessed the impact of the training curriculum on each participant’s self-perceived ability to perform his/her duties.

## METHODS

### Study Design and Setting

This pre-post study was conducted in Luanda, Angola. A conference room providing space for presentations, breakout sessions, and simulation were used during the two weeks of training in the hospital. Three native Portuguese speakers provided direct interpretation when needed during the two weeks of training. All educational materials including presentations were translated by native Portuguese speakers prior to the event. The planning team from the Angolan military and US Navy met prior to the event and used previous curriculums to establish the training topics, activities, and schedule for this specific program.

A survey was provided to the 32 study participants before the initiation of training and immediately upon completion of the training. The pre-event survey included six Likert-scale questions assessing the individuals’ perceived ability to work and manage a real sample in a contagious environment and interpret the results. The post-survey questionnaire included these same six questions, two additional Likert-scale questions assessing the overall effectiveness of the training, and a ninth open-ended question requesting comments for needed additional training. The study design used SQIRE 2.0 guidelines for quality improvement reporting.^9^ The study received a waiver from the institutional review board.

### Selection of Participants

A total of 32 Angolan classroom participants completed the course and the surveys. Chosen by the Forças Armadas Angolanas (FAA) and the Angolan MOH, the participants were a mixture of civilian and military physicians, nurses, social workers, and lab technicians. This program was sponsored by the US Africa Command (US AFRICOM).

### Intervention

A pre-course survey was provided to the 32 study participants before the initiation of training and immediately upon completion of the training. The pre-intervention survey included six Likert-scale questions assessing their perceived ability to handle and manage a real sample in a contagious environment, and independently interpret the results. The first week’s training (December 2–6, 2019) covered topics and training that the planning teams identified as high-yield prior to the training to include public health, disaster response, donning/doffing of PPE, and patient and Ebola treatment unit protocols. The second week of training (December 9–13, 2019) focused on lab concepts that included the following: basic lab skills; lab safety, setup and use of the mobile lab; and RT-PCR skills. The majority of the participants had little to no prior experience with this equipment prior to the CHERRT training.

The post-intervention questionnaire included the same six pre-intervention questions, two additional Likert-scale questions assessing the overall effectiveness of the training, and a final ninth question requesting comments for needed additional training (Table 1).

### Methods of Measurement and Outcome Measures

The primary outcome was the calculated change in the six Likert-scored questions asked before and after the training assessing self-perceived competence and ability to perform their respective duties on the team. These six questions were provided on an anonymous form in Portuguese using a 7-point Likert scale ranging from “1,” designated as strongly disagree, to “7,” designated as strongly agree. Translators were available in Portuguese to assist with questions on the survey. The participants were instructed to circle one number from one to seven for each of the Likert-scale questions. Each participant was given a unique identifier allowing for anonymity and pairing analysis. The survey abstractors were not blinded to the study hypothesis.

Secondary outcomes included the two additional Likert-scale questions assessing overall effectiveness of the training and a final, open-ended comment section eliciting recommendations for additional training that the respondent felt was needed or desired. The two additional post-intervention questions used the same 7-point Likert scale as the pre-intervention assessment, allowing for consistency.

### Primary Data Analysis

The completed survey data was entered into Microsoft Office Excel 2007 (Microsoft Corp, Redmond, WA). We calculated means and standard deviations (SD) for each of the six pre- and post-intervention questions that were repeated on the surveys for comparison, and the two questions that were only asked on the post-intervention questionnaire. The comments elicited from the final question were translated into English by a professional translator identified by the US Armed Forces team. Using the unique identifiers, we compared the six repeated pre- and post-self-assessment questions using a paired t-test. Means, SDs, 95% confidence intervals (CI), and two-tailed p values were calculated for each of the six questions. We also calculated means and SDs for the two unique post-intervention questions regarding participants’ assessment of the training program.

A total of 32 pre-intervention and 27 post-intervention questionnaires were completed. Five individuals did not participate during the last day of the program that included ceremonial activities and completion of the survey. These five individuals were considered lost to follow-up. The pre- and post-intervention surveys were paired using the unique identifiers.

## RESULTS

All six of the questions – 1) “I feel confident managing a real laboratory sample test for Ebola or other highly contagious sample” (95% CI, −3.53 to −1.65; p=<0.0001); 2) “I feel safe working in the lab environment during a real scenario” (95% CI, −3.64 to −1.59; p=<0.0001); 3) “I feel as if I can appropriately manage a potentially highly contagious laboratory sample” (95% CI, −3.90 to −2.17; p=<0.0001); 4) “I feel that I can interpret a positive or negative sample during a suspected contagious outbreak” (95% CI, −2.12 to −0.40; p=0.006), 5) “I understand basic Biobubble/mobile laboratory concepts and procedures”(95% CI, −4.69 to −0.79; p=<0.0001); and 6) “I understand PCR principles” (95% CI, −3.17 to −1.31; p=<0.0001) – showed statistically significant change pre and post training (Table 1). The course participants scored highly on the final two post-training questions – “I can more effectively perform my role/position because of the training I received during this course” (6.74), and “This training was valuable” (7.00).

The participants were provided a final open-ended question: “What additional training is needed or desired?” Comments from this question included requests for more hands-on time, epidemiology, prehospital and general patient transport, additional disease review, more frequent training for skill and knowledge maintenance, additional statistics on disease impact, and organizational communication.

## DISCUSSION

As of March 5, 2020, WHO reported 1,133,758 confirmed cases and 62,784 confirmed coronavirus (COVID-19) deaths affecting 204 countries/regions.^1^ Drawing parallels to the West African Ebola outbreak in 2014 that infected over 20,000 individuals resulting in approximately 11,000 deaths,^4^,^9^ the Ebola outbreak provides many lessons for future epidemics/pandemics, such as the current COVID-19 outbreak. These lessons learned include a need for increased surveillance, more effective ecological health interventions, expanded prediction modeling and improved risk communication, as well as improved diagnostic tools, medications and vaccines, and local and global response.^10^ Interventions created and employed around the world to respond to highly infectious disease outbreaks include RRTs and a mobile lab for rapid, on-scene diagnostics. The Angolan RRT is trained to respond to an infection of public concern as well as larger concepts of disaster response and management that will help contain the spread of any potentially infectious disease outbreak. The nation of Angola with the assistance of the US Armed Forces identified 32 individuals of various specialties with a focus on lab personnel for this annual CHERRT training.

The WHO EVD Consolidated Preparedness Checklist, Revision 1,^8^ identifies 11 key components requiring minimal resources, and was used as a baseline for the training competencies. These competencies were supplemented with protocols, checklists, standard operating procedures (SOP), and instructor experiences. The team’s training on these concepts as assessed by the pre- and post-course intervention showed statistically significant changes in all six categories. These scores of self-perceived improved abilities, knowledge, and confidence provide evidence that this type of training improves personnel’s perception in the team’s ability to respond based on the training experience.

For COVID-19, as of March 2020 the lab capacity in the US did not meet the need for diagnostics testing even after the entry of commercial lab companies such as Laboratory Corporation of America Holdings and Quest Diagnostic.^11^ Improving lab capacity is important in assessing the extent of the outbreak in the US as well as in LMICs on the continent of Africa, which has limited diagnostic resources. The mobile lab is one such resource that is readily deployable, cost effective, and provides a safe containment platform for rapid on-scene diagnostic capabilities. The sampling and testing methods are not dependent on shipping the samples to a reference lab, thus speeding up diagnostic turnaround in LMICs or regions of the world that lack such labs. The current pandemic has identified an additional gap with regard to labs. It seems that even when labs are available, they can be quickly overwhelmed by the increased number of samples. This proposed model could be easily implemented with confidence and minimal training based on accepted protocols and past experience.

Working in highly contagious and volatile environments requires confidence in one’s abilities as well as knowledge of the situation and environment (situational awareness). This confidence translates to an awareness of the environment inside a soft-walled lab with biocontainment capability, reducing the risk of cross-contamination of samples and/or spillage. Formal training based on lessons learned, consensus protocols and checklists, and provider experience provide a foundation for adequately trained teams that can effectively intervene and contain a global outbreak.

A long-term follow-up of the participants’ abilities, as well as an assessment of each future activation, may further strengthen the perceived benefits of this training curriculum. The pre and post assessments, survey statements, participants’ comments, and national/regional priorities provide the material for continued adjustments on curriculum development and implementation.

## LIMITATIONS

There are several limitations to this study. First, we often had to use materials translated from English to Portuguese. Native Portuguese speakers from the US Armed Forces were used for translation of instructional materials, surveys, and presentations when needed for clarification. Additionally, some of the participants took part in prior disaster training and/or Ebola workshops three years prior to this intervention. This earlier training was provided by the lead instructor; therefore, some program participants already had some baseline knowledge of the presented material. To that extent, in preparation for the final workshop, the RRT conducted some of its own training prior to the engagement.

The survey focused on the specific training event; however, prior training or lab experience by some individuals could potentially have contributed to the relatively higher scores on the pre-intervention questions that did not achieve significance. The team was hand-picked by the MOH and the military, allowing for potential selection bias for better trained and educated personnel. Additionally, the survey abstractors were not blinded to the study hypothesis. The assessments are based on self-reported competence and ability after the training and simulations and do not reflect actual events. Finally, this study’s limitations include those inherent to any pre- and post-intervention survey methodology.

## CONCLUSION

This Angolan CHERRT training curriculum based on WHO guidelines, After Action Reports, a NIH standard mobile lab curriculum, and internationally accepted standard operating procedures, provides the nation of Angola with the confidence to rapidly respond at the national level to a highly infectious contagion in the region and perform onsite mobile lab diagnostics. This mobile RRT laboratory provides a potential rapid diagnostic resource when epidemic/pandemic resource allocation may need to be prioritized based on confirmed disease prevalence.

## Figures and Tables

**Table 1:** Pre- and post-course survey questions administered to participants who underwent training in the use of mobile labs and on-site diagnostic testing.

Question	Pre-course mean ± SD (n = 32)	Post-course mean ± SD (n =27)	P-value
1. I feel confident managing a real laboratory sample test for Ebola or other highly contagious sample.	3.56 ± 2.28	6.15 ± 1.32	<0.0001
2. I feel safe working in the lab environment during a real scenario.	3.85 ± 2.56	6.46 ± 1.24	<0.0001
3. I feel as if I can appropriately manage a potentially highly contagious laboratory sample.	3.00 ± 2.14	6.04 ± 1.43	<0.0001
4. I feel that I can interpret a positive or negative sample during a suspected contagious outbreak.	3.15 ± 2.28	4.41 ± 2.42	0.006
5. I understand basic Biobubble/mobile laboratory concepts and procedures.	2.74 ± 2.12	6.48 ± 1.48	<0.0001
6. I understand PCR (polymerase chain reaction) principles.	2.92 ± 2.12	5.16 ± 1.84	<0.0001
7. I can more effectively perform my role/position because of the training I received during this course.		6.74	
8. This training was valuable.		7.00	

*SD*, standard deviation.

Scores based on 1 to 7 Likert scale. 1 = Strongly Disagree, 4 = Neutral, 7 = Strongly Agree.
